# From resistant hypertension to renal denervation: an emerging therapeutic approach in light of new international guidelines

**DOI:** 10.1016/j.ijcrp.2025.200550

**Published:** 2025-11-17

**Authors:** Elena-Mihaela Cordeanu, Emma Morisot, François Bronner, Eric Prinz, Dominique Stephan

**Affiliations:** a Service d'HTA et maladies vasculaires, Hôpitaux Universitaires de Strasbourg, France; b Service de Cardiologie, Hôpitaux Universitaires de Strasbourg, France; c Service de Néphrologie, Hôpitaux Universitaires de Strasbourg, France

**Keywords:** Resistant hypertension, Renal denervation, Sympathetic nervous system, Percutaneous intervention, European guidelines, Blood pressure control, Cardiovascular risk prevention, Device-based therapy, Radiofrequency ablation, Ultrasound ablation

## Abstract

**Background:**

Resistant hypertension (RH) remains a major therapeutic challenge, affecting 12–18 % of treated hypertensive patients and associated with increased cardiovascular risk. Renal denervation (RDN) has emerged as a promising therapeutic option following initially mixed results.

**Objective:**

This review analyzes the evolution of RH management, from its definition to new therapeutic perspectives offered by RDN, based on recent international guidelines (ESH 2023, ESC 2024, ACC/AHA 2025).

**Methods:**

Critical analysis of pivotal clinical trials, recent meta-analyses, and international registries evaluating the efficacy and safety of RDN in RH.

**Results:**

Recent trials (SPYRAL HTN-ON/OFF MED, RADIANCE-HTN TRIO) demonstrate moderate but significant efficacy of RDN, with systolic blood pressure reductions ranging from 3.9 to 18.7 mmHg depending on populations. Technological advances (multi-electrode catheters, distal branch targeting) improve outcomes. The safety profile appears favorable with low complication rates.

**Conclusions:**

RDN represents an emerging therapeutic option for RH, now recommended by European societies for specific indications. Rigorous patient selection and performance in experienced centers remain essential.

## 1 Introduction

Hypertension represents the leading modifiable cardiovascular risk factor worldwide, affecting over one billion individuals and constituting a major public health challenge [1]. Despite considerable progress in understanding its pathophysiology and the continuous enrichment of the therapeutic arsenal, a significant proportion of patients remain poorly controlled, defining what we term resistant hypertension (RH). This particular clinical entity, affecting 12–18 % of treated hypertensive patients, not only resists conventional treatments but is also associated with a poor cardiovascular prognosis, including increased incidence of stroke, myocardial infarction, and heart failure [2]. Face with the limitations of optimal medical therapy and the therapeutic impasse that RH sometimes represents, an innovative interventional approach has emerged over the past decade: renal denervation (RDN) [3]. This technique, which aims to interrupt perivascular renal sympathetic nerve fibers, is based on a thorough understanding of the pathophysiological mechanisms of resistant hypertension. The recent evolution of the European regulatory landscape, with the publication of guidelines from the European Society of Hypertension (ESH) in 2023 [4]and the European Society of Cardiology (ESC) in 2024 [5], marks a decisive turning point. These scientific societies now integrate RDN into the therapeutic options for RH, thereby recognizing its legitimate role in managing patients who remain refractory to optimal medical therapy.

### 1.1 Resistant hypertension: definition and contemporary challenges

The classic definition of resistant hypertension, while seemingly simple, masks a complex clinical reality. According to established criteria, RH is characterized by uncontrolled blood pressure beyond therapeutic targets, despite concomitant administration of at least three antihypertensive agents from different classes at optimal doses, generally including a calcium channel blocker, a renin-angiotensin system blocker, and a thiazide diuretic [4,5]. This definition, while having the advantage of clarity, is now subject to criticism from the scientific community. The main limitations identified reflect the intrinsic complexity of this pathology. The variability of blood pressure thresholds according to international guidelines (≥140/90 mmHg versus ≥130/80 mmHg) complicates the evaluation of prevalence and associated risk. The absence of consensus on the number of BP measurements or the duration necessary before establishing the diagnosis introduces diagnostic uncertainty. More fundamentally, the current definition does not distinguish the inability to achieve normal blood pressure from the impossibility of reaching personalized therapeutic targets, thus omitting contextual factors such as age, comorbidities, or treatment tolerance [2,4,5]. The pathophysiology of RH reveals the complex interaction of several interdependent mechanisms whose understanding is crucial for considering innovative therapeutic approaches. Sympathetic nervous system hyperactivity represents a key component of these interconnected mechanisms, with particular prominence in young and obese subjects [6]. This hyperactivity manifests as excessive vasoconstriction, increased cardiac output, and enhanced renal sodium reabsorption. Concurrently, sodium retention and volume expansion are particularly prevalent in salt-sensitive patients, especially those of African or Asian ancestry, where excessive dietary sodium intake perpetuates elevated blood pressure [6]. Finally, arterial stiffness and vascular remodeling, particularly observed in elderly patients with isolated systolic hypertension, complete this complex pathophysiological picture and often explain resistance to conventional therapeutic approaches [7]. The prognostic impact of RH cannot be underestimated. Beyond elevated blood pressure figures, the overall cardiovascular risk is significantly increased. Data from the international Global SIMPLICITY registry are particularly compelling in this regard: they reveal a three-year major adverse cardiovascular event (MACE) rate of 10.3 % in poorly controlled patients, compared to only 2.9 % in those whose blood pressure is well managed [8]. This considerable difference underlines the crucial importance of optimal blood pressure control and justifies the search for innovative therapeutic approaches when conventional treatments reach their limits.

### 1.2 Renal denervation: concept and technological evolution

**Fig. 1:**
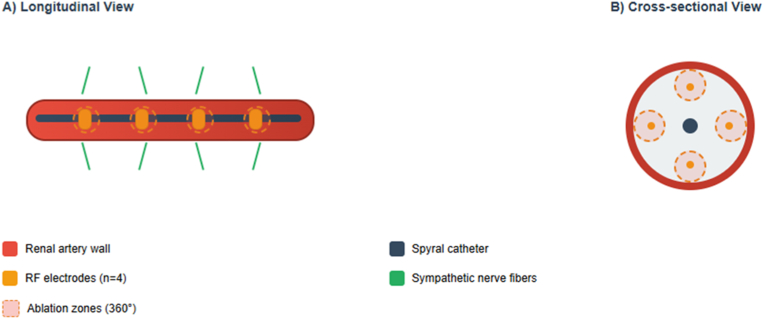
Symplicity Spyral Multi-electrode renal denervation system. (A) Longitudinal and (B) cross-sectional views of the four-electrode catheter system. Radiofrequency ablation creates 360° circumferential zones targeting perivascular sympathetic nerve fibers (green lines). Dashed circles indicate ablation zones. Note: This is an original figure created by the authors based on publicly available design schematics (Medtronic Symplicity Spyral). (For interpretation of the references to colour in this figure legend, the reader is referred to the Web version of this article.)

RDN draws its foundations from a thorough understanding of the role of the sympathetic nervous system in blood pressure regulation. The renal sympathetic nervous system indeed occupies a strategic position in this regulation, modulating three fundamental mechanisms: stimulation of renin release, modulation of renal vascular tone, and regulation of sodium excretion [9]. Under physiological conditions, renal sympathetic nerves maintain homeostatic control of BP by dynamically adjusting renal blood flow, tubular sodium reabsorption, and renin secretion [10]. Efferent sympathetic fibers, originating from the celiac and aorticorenal ganglia, exert three primary effects: stimulation of renin secretion from juxtaglomerular cells via β1-adrenergic receptors, direct vasoconstriction of afferent and efferent arterioles through α1-adrenergic activation, and enhancement of proximal and distal tubular sodium reabsorption, collectively elevating systemic vascular resistance and extracellular volume [[11], [12], [13]]. Conversely, renal afferent sensory fibers, located predominantly in the renal pelvic wall, transmit mechanosensitive and chemosensitive signals from the kidney via dorsal root ganglia (T10-L1) to central autonomic centers, including the paraventricular nucleus, rostral ventrolateral medulla, and nucleus tractus solitarii, modulating overall sympathetic outflow [[14], [15], [16]]. In pathological states such as resistant hypertension, chronic renal sympathetic hyperactivity disrupts this homeostatic balance, establishing a positive feedback loop characterized by sustained renin-angiotensin system activation, impaired pressure-natriuresis, and progressive volume expansion that perpetuates elevated blood pressure [[16], [17], [18]]. Hyperactivity of this system, frequently observed in resistant hypertension, contributes to chronic blood pressure elevation by stimulating the renin-angiotensin-aldosterone system, promoting sodium and water retention, and inducing persistent vasoconstriction [6]. The principle of RDN therefore relies on targeted interruption of these sympathetic nerve fibers located in the adventitia of renal arteries, with the objective of reducing this deleterious hyperactivity and restoring physiological blood pressure regulation [19]. Pharmacological and interventional approaches modulate sympathetic activity through distinct mechanisms. Antihypertensive agents such as β-blockers and centrally acting sympatholytics like moxonidine produce systemic, reversible inhibition of sympathetic tone by modulating neurotransmitter release or receptor responsiveness at the level of end-organ receptors [10,[20], [21], [22]]. In contrast, RDN achieves direct, localized, and durable ablation of both afferent and efferent renal sympathetic fibers within the arterial adventitia, thereby attenuating both peripheral sympathetic signaling to the kidney and central sympathetic outflow mediated by renal afferent pathways [10,16]. Importantly, pharmacological therapies act downstream of pathological renal sympathetic hyperactivity by blocking intermediate signaling pathways, whereas RDN targets the proximal source of sympathetic dysregulation [11]. These strategies are complementary rather than competitive: pharmacological therapy provides titratable systemic control, while RDN offers sustained, kidney-specific neuromodulation that persists independent of medication adherence in selected patients. This conceptual approach, appealing in its pathophysiological logic, has been validated by numerous preclinical studies that confirmed the absence of functional nerve regrowth in the long term after radiofrequency ablation, thus guaranteeing the durability of the therapeutic effect [23]. The technological evolution of RDN reflects the constant search for optimized efficacy and maximum safety [24]. Two technological modalities currently dominate the RDN field, each with its specificities and advantages. Radiofrequency, the historical approach to denervation, relies on delivering thermal energy via electrodes in direct contact with the arterial wall [25]. The major evolution of this technique lies in the development of multi-electrode catheters, of which the Symplicity Spyral system from Medtronic constitutes the most accomplished example [26]. This innovative device presents a unique design allowing automatic positioning of four electrodes, thus performing circumferential ablations at 360° in four distinct quadrants (Fig. 1). This approach guarantees a consistent and reproducible ablation pattern, optimizing treatment efficacy while preferentially targeting distal branches of the renal artery, an area where sympathetic innervation density is maximal [27]. The technological alternative represented by ultrasound presents distinct characteristics that make it an interesting complementary approach. The Paradise system uses a liquid-filled balloon that diffuses ultrasound energy at 360° without direct contact with the vascular wall [28]. This technology allows uniform distribution of ablation throughout the entire vascular circumference and presents the theoretical advantage of preserving endothelial integrity, potentially reducing the risk of vascular complications.

### 1.3 Clinical evidence evolution: from initial promises to current renaissance

Initial enthusiasm for RDN was rapidly fueled by the spectacular results of the SYMPLICITY HTN-2 trial in 2010, which reported a remarkable reduction of −32 mmHg in systolic blood pressure at six months in the denervation treatment group, compared to an increase of +1 mmHg in the control group [29]. The French DENERHTN study, published in 2015, provided welcome confirmation of RDN efficacy in a more rigorous methodological framework [30]. This randomized controlled trial demonstrated in 106 patients with RH a significant reduction in daytime ambulatory systolic blood pressure of −15.8 mmHg in the RDN group versus −9.9 mmHg in the control group. However, these pioneering studies presented methodological limitations that were subsequently identified, including the absence of a sham procedure, the lack of mandatory 24-h ambulatory blood pressure monitoring to exclude white-coat hypertension and limited follow-up duration, which may have contributed to overestimation of treatment effects. The turning point occurred in 2014 with the publication of results from the SYMPLICITY HTN-3 trial, an American study of exemplary methodological accuracy [31]. Randomized, sham-controlled, and conducted double-blind, this trial showed no significant difference between the denervation group and the control group at six months, questioning the efficacy of the procedure and causing a marked slowdown in its clinical adoption. More importantly, potential safety concerns and procedural complications became apparent as RDN gained wider clinical adoption and real-world experience accumulated [32,33]. This period of disillusionment, while challenging for the research community, proved beneficial for scientific advancement, prompting comprehensive analysis of the underlying causes of this apparent failure [34,35]. The renaissance of RDN emerged through a new generation of clinical trials that incorporated lessons learned from previous methodological limitations. The SPYRAL HTN-ON MED trial, using a new device, published in 2022, included hypertensive patients maintained on their stable antihypertensive treatment, approaching real clinical practice conditions [36]. The results showed a reduction in ambulatory systolic pressure of −18.7 mmHg in the denervation group versus −8.6 mmHg in the group that underwent a sham procedure. Simultaneously, the SPYRAL HTN-OFF MED trial, conducted in untreated hypertensive patients, demonstrated more modest but significant results with a reduction of −3.9 mmHg in ambulatory systolic pressure versus placebo [37], confirming the procedure's specific efficacy. The European RADIANCE-HTN TRIO trial, published in 2021, represents an important step in this renaissance by evaluating for the first time the efficacy of ultrasound RDN in a randomized trial controlled by sham procedure [38]. This study, including 136 poorly controlled hypertensive patients on triple antihypertensive therapy, demonstrated at two months a significant reduction in ambulatory systolic pressure of −8.0 mmHg in the denervation group versus −3.0 mmHg in the sham group, with a statistically significant adjusted difference of −4.5 mmHg. Additionally, the RADIANCE II trial confirmed the efficacy of ultrasound RDN in a broader population of hypertensive patients [39]. The international Global SIMPLICITY registry, including more than 3000 patients across 18 countries, provided valuable real-world evidence on RDN efficacy and safety [40]. Data from this registry reveal encouraging results with an average reduction of −11.6 mmHg in office systolic pressure and −6.6 mmHg in ambulatory pressure at six months [41]. Particularly remarkable are the results obtained in patients with severe hypertension, where blood pressure reductions reach −20.3 mmHg and −8.9 mmHg respectively for office and ambulatory systolic pressures. The variability in blood pressure reduction observed across studies, ranging from approximately 3.9 to 18.7 mmHg, likely reflects heterogeneity in study design and patient characteristics [36,37]. Key contributors include baseline BP severity, degree of sympathetic activation, medication burden, procedural completeness, and anatomical factors such as renal nerve density and nerve-to-lumen distance [42,43]. Patient-related factors, including age, body mass index, arterial stiffness, and medication adherence, further influence outcomes [44]. Notably, post-hoc analyses from the Global SYMPLICITY DEFINE registry demonstrate that patients with severe baseline hypertension (systolic BP ≥ 180 mmHg) exhibit significantly greater absolute BP reductions compared with those with moderate elevations, with mean reductions approaching 20 mmHg in this subgroup [45,46]. These findings emphasize the importance of rigorous patient phenotyping and procedural standardization in optimizing therapeutic efficacy. Table 1 summarizes the pivotal clinical trials that shaped the evolution of RDN from initial promises to current evidence-based practice.

**Table 1:** Clinical evidence evolution: key renal denervation trials.

Study	Year	Design	Population	Technology	Primary Endpoint	RD Group	Control Group	Treatment Difference	Ref
**SYMPLICITY HTN-2**	2010	RCT, open-label	Treatment-resistant HTN	RF (Flex)	Office SBP at 6 months	−32 mmHg	+1 mmHg	−33 mmHg	[29]
**DENERHTN**	2015	RCT, open-label	Resistant HTN (n = 106)	RF (Flex)	Daytime ambulatory SBP	−15.8 mmHg	−9.9 mmHg	−5.9 mmHg	[30]
**SYMPLICITY HTN-3**	2014	RCT, sham-controlled	Treatment-resistant HTN	RF (Flex)	Office SBP at 6 months	No significant difference vs sham	–	NS	[31]
**SPYRAL HTN-ON MED**	2022	RCT, sham-controlled	HTN on medications	RF (Spyral)	24-h ambulatory SBP	−18.7 mmHg	−8.6 mmHg	−10.1 mmHg	[36]
**SPYRAL HTN-OFF MED**	2020	RCT, sham-controlled	HTN without medications	RF (Spyral)	24-h ambulatory SBP	−3.9 mmHg vs placebo	–	−3.9 mmHg	[37]
**RADIANCE-HTN TRIO**	2021	RCT, sham-controlled	HTN on triple therapy (n = 136)	Ultrasound	24-h ambulatory SBP	−8.0 mmHg	−3.0 mmHg	−4.5 mmHg	[38]
**RADIANCE II**	2023	RCT, sham-controlled	Broader HTN population	Ultrasound	24-h ambulatory SBP	Confirmed efficacy	–	Significant	[39]
**GLOBAL SIMPLICITY Registry**	2015-ongoing	Registry, real-world	>3000 patients, 18 countries	RF (various)	Multiple endpoints	Office: 11.6 mmHg<br>Ambulatory: 6.6 mmHg	–	–	[40,41]
**GLOBAL SIMPLICITY - Severe HTN**	2020	Registry subset	Severe hypertension	RF (various)	Blood pressure reduction	Office: 20.3 mmHg<br>Ambulatory: 8.9 mmHg	–	–	[41]
**GLOBAL SIMPLICITY - 36 months**	2023	Registry follow-up	Long-term cohort	RF (various)	Durability	Sustained BP reduction	–	–	[46]

**Abbreviations:** RCT: Randomized controlled trial; HTN: Hypertension; SBP: Systolic blood pressure; RD: Renal denervation; NS: Not significant; BP: Blood pressure.

The durability of these effects, confirmed by 36-month follow-up showing persistence of blood pressure reductions, reinforces the long-term interest of this therapeutic approach [46]. The time course of BP reduction following RDN demonstrates characteristic biphasic kinetics with important clinical implications. Sham-controlled trials consistently document initial BP decline within 2–4 weeks post-procedure, progressive accentuation over 1–3 months, and a sustained plateau thereafter [29,[36], [37], [38], [39]]. Long-term registry data extending to 36 months confirm maintenance of antihypertensive efficacy without attenuation [24,46] with recent 9-year follow-up demonstrating persistent blood pressure reductions and absence of functional nerve regeneration [47]. Table 2 summarizes the temporal evolution of BP response across key studies, highlighting both the early onset and long-term persistence of treatment benefit.

**Table 2:** Temporal evolution of systolic blood pressure (SBP) reduction after renal denervation in major trials.

Study	Baseline SBP (mmHg)	Timepoint	Office SBP change (mmHg)	Ambulatory SBP change (mmHg)	Reference
**SYMPLICITY HTN-2**	178	1 mo/6 mo	−20/−32	−12/−15	[29]
**SPYRAL HTN-ON MED**	165	3 mo/6 mo/12 mo	−10/−18/−17	−6/−9/−8	[36]
**SPYRAL HTN-OFF MED**	162	3 mo/6 mo	−4/−6	−3/−4	[37]
**RADIANCE-HTN TRIO**	145^a^	2 mo/6 mo/12 mo	−8/−10/−10	−4/−6/−6	[38]
**RADIANCE II**	151^a^	3 mo/6 mo/12 mo	−7/−9/−10	−4/−6/−6	[39]

a Daytime ambulatory SBP baseline; SBP: systolic blood pressure; values represent mean changes from baseline in the RDN group.

### 1.4 Safety profile and clinical considerations

**Fig. 2:**
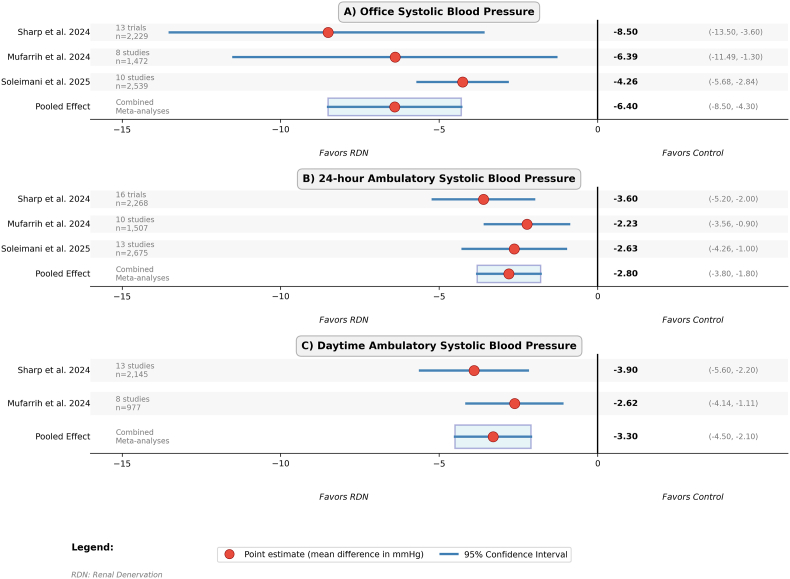
Meta-analyses of blood pressure reductions after renal denervation. Forest plot showing consistent modest but statistically significant reductions across different measurement modalities, with larger effects observed in office compared to ambulatory measurements [3,52,53].

The evaluation of the RDN safety profile constitutes a major issue for the acceptability of this interventional technique. Data accumulated over the past decade paint a globally reassuring picture, with low complication rates and generally minor and transient adverse events [33,[48], [49], [50]]. The most frequently reported complications include vascular events at the femoral puncture site, such as hematomas or pseudoaneurysms, usual complications of any percutaneous procedure and generally without severity [51]. Renal arterial lesions, a theoretical safety concern to their potential impact on kidney function, remain exceptional and mainly include minor stenoses or dissections, most often asymptomatic and without functional consequence [49]. Preservation of kidney function naturally represents a central concern during any intervention on renal arteries. In this regard, available data are particularly reassuring. An exhaustive systematic review published in 2017, grouping 52 quantitative studies and 14 qualitative studies for 2898 patients, showed no significant deterioration in kidney function up to nine months after the procedure [49]. This absence of negative impact on kidney function is confirmed by a more recent meta-analysis published in 2020, which evaluated RDN safety in 50 clinical trials representing more than 5700 patients and 10,000 patient-years of follow-up [33]. Long-term safety data extending up to 9 years post-procedure continue to demonstrate sustained blood pressure reductions without adverse effects on kidney function [47]. Recent meta-analyses confirm moderate but statistically significant efficacy of RDN on blood pressure reduction. A comprehensive systematic review and meta-analysis published in 2024 in the Journal of Hypertension, including 25 randomized controlled trials, reported significant reductions of −8.5 mmHg for office systolic BP, −3.6 mmHg for 24-h ambulatory systolic BP, and −3.9 mmHg for daytime ambulatory systolic BP in favor of RDN [3]. Multiple recent meta-analyses have consistently demonstrated the efficacy of RDN, with a 2025 systematic review specifically focusing on sham-controlled trials reporting similar blood pressure reductions [52]. Another comprehensive meta-analysis published in the Journal of the American Heart Association confirmed these findings across different patient populations and device types [53]. This reduction, although moderate in absolute value, carries considerable clinical importance given epidemiological data establishing that a reduction of only 5 mmHg in systolic pressure is associated with a 14 % decrease in stroke risk and 9 % in coronary heart disease risk [54] (Fig. 2). The accumulation of consistent clinical and registry evidence has prompted renewed evaluation of RDN within international hypertension guidelines.

### 1.5 New european guidelines: a paradigm shift

The evolution of the European regulatory landscape concerning RDN marks a decisive turning point in the official recognition of this therapeutic option. The European Society of Hypertension (ESH) guidelines published in 2023 integrated RDN for the first time into the therapeutic armamentarium for resistant hypertension assigning it an acceptable level of recommendation and evidence [4]. The ESH recommends that RDN may be considered as a therapeutic option for patients with an estimated glomerular filtration rate (eGFR) ≥40 mL/min/1.73 m^2^ and whose blood pressure remains uncontrolled despite antihypertensive polytherapy, with a class IIb recommendation and level B evidence [4]. The guidelines also specify that RDN may be considered as a complementary therapeutic option in patients suffering from resistant hypertension even in cases of more impaired kidney function, down to an eGFR threshold of 15 mL/min/1.73 m^2^. This extension of the indication testifies to growing confidence in the procedure's safety profile, even in higher-risk patients. ESH additionally emphizes the necessity of a shared decision-making process between the clinicians and patients, recognizing the complexity of this therapeutic decision and the importance of patient adherence to the proposed treatment.

**Fig. 3:**
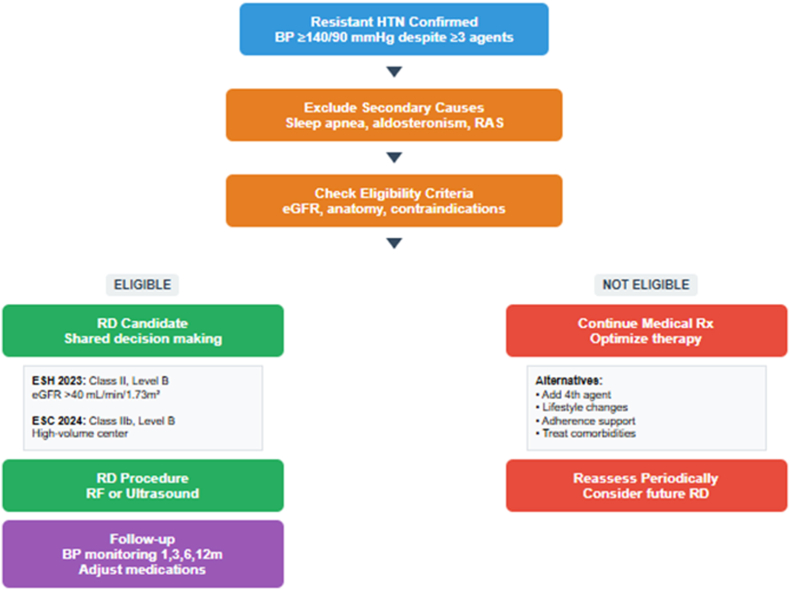
Clinical decision algorithm for renal denervation. The flowchart guides clinicians from resistant hypertension diagnosis through patient selection, procedural decision-making and post-procedural follow-up, incorporating current European guidelines (ESH 2023, ESC 2024) and American recommendations (AHA 2024). Eligible patients undergo renal denervation with structured follow-up, while non-eligible patients continue optimized medical therapy with periodic reassessment.

The European Society of Cardiology, in its 2024 guidelines, adopted a slightly more conservative yet clearly favorable position [5]. These guidelines recommend that RDN may be considered, in medium-to high-volume centers for patients whose blood pressure remains uncontrolled despite a combination of three antihypertensive agents including a thiazide or thiazide-like diuretic, with a class IIb recommendation and level B evidence. Although more restrictive than the ESH, this position consolidates RDN's place as a recognized adjunctive therapy within the antihypertensive arsenal. In the United States, the regulatory trajectory followed a temporal delay rather than a conceptual divergence. After the 2023 FDA approval of RDN systems (ultrasound- and radiofrequency-based), the American Heart Association (AHA) scientific statement on RDN from 2024 provided a comprehensive review of the evidence, emphasizing the importance of proper patient selection and procedural expertise [55]. While previousACC/AHA hypertension guidelines did not include specific recommendations for RDN, [56], the 2025 AHA/ACC hypertension guideline formally integrated RDN into clinical practice with a class IIb, level B-R recommendation, stating that the procedure may be reasonable in carefully selected adults with resistant hypertension (office SBP 140–180 mmHg, DBP ≥90 mmHg, eGFR ≥40 mL/min/1.73 m^2^) who remain uncontrolled despite optimal therapy or are intolerant to further drug escalation [57]. The new guideline thus closes the two-year gap with European societies, aligning the American position with contemporary evidence and real-world experience rather than expressing greater caution.Table 3 provides a comprehensive comparison of current international guidelines regarding RDN, highlighting the evolving landscape of recommendations and the differences between European and American approaches. Fig. 3 presents a practical clinical decision algorithm for implementing RDN in routine practice, incorporating the key steps from initial hypertension diagnosis through post-procedural follow-up.

**Table 3:** International guidelines comparison for renal denervation.

	ESH 2023	ESC 2024	AHA 2024^a^	AHA/ACC/AAPA 2025
**Recommendation Class**	IIb	IIb	Not graded (scientific overview only)	Class IIb
**Evidence Level**	B	B	–	B-R
**Primary Indication**	Resistant HTN with uncontrolled BP despite ≥3 drugs (including diuretic)	Uncontrolled HTN despite ≥3 antihypertensive agents (including thiazide/thiazide-like)	Uncontrolled or resistant HTN despite polytherapy or intolerance (evidence reviewed only)	Adults with office SBP 140–180 mmHg and DBP ≥90 mmHg on maximally tolerated therapy or unable to escalate further
**Blood Pressure Criteria**	Uncontrolled BP ≥ 140/90 mmHg despite antihypertensive polytherapy	Uncontrolled BP despite combination of 3 antihypertensive agents	Not specified	Office SBP 140–180 mmHg, DBP ≥90 mmHg
**Medication Requirements**	≥3 antihypertensive agents (including thiazide or thiazide-like)	≥3 antihypertensive agents (including thiazide or thiazide-like)	Not specified	On stable optimized triple therapy ± MRA or intolerant to further drug escalation
**eGFR Threshold (primary)**	≥40 mL/min/1.73m^2^	≥40 mL/min/1.73m^2^	Not explicitly stated but trials excluded <40 mL/min	≥40 mL/min/1.73 m^2^
**eGFR Threshold (extended)**	>15 mL/min/1.73m^2^ for resistant HTN (conditional expert consensus)	<40 mL/min → not recommended (Class III C)	Not specified	avoid <40 mL/min pending data
**Center Requirements**	High-volume/experienced centers recommended for procedural safety	Medium to high volume centers (Class IIb B)	Experienced centers (emphasized but not graded)	Experienced centers with multidisciplinary team (Class I B)
**Decision-Making**	Shared decision-making process with patient and team emphasized	Mandatory shared risk–benefit discussion and multidisciplinary assessment	Proper patient selection and informed consent emphasized	Shared decision-making required before procedure (Class I C)
**Patient Population**	Broad resistant HTN population (« true resistance »)	More restrictive criteria (confirmed resistance and patient preference)	Selected patients with resistant and moderate HTN (narrative data)	Resistant HTN or intolerance to drugs
**Regulatory Status**	Endorsed with recommendations (CE-marked devices endorsed in Europe)	Cautiously endorsed	Based on FDA approval (2023) of Paradise & Spyral systems	Fully integrated into AHA/ACC guideline algorithm as adjunct therapy
**Geographic Scope**	Europe	Europe	U.S. scientific statement	U.S. clinical guideline
**Year of Publication**	2023	2024	2024	2025
**Approach**	More inclusive	More conservative	Educational/scientific overview	Formal clinical endorsement within treatment pathway

BP: blood pressure; B-R = moderate-quality evidence from RCTs; DBP: diastolic blood pressure; eGFR: estimated glomerular filtration rate; FDA: Food and drug administration; HTN: hypertension; MRA: mineralocorticoid receptor antagonists; SBP: systolic blood pressure; U.S.: United States.

Notes.

a AHA 2024 refers to the scientific statement on renal denervation, not formal clinical practice guidelines.

### 1.6 Future perspectives and clinical implementation

The future of RDN is articulated around several development axes that condition its evolution toward a mature and widely accepted therapeutic technique. Optimal patient selection probably represents the most important challenge to address in the coming years [58]. Identification of predictive response biomarkers, whether related to sympathetic nervous system activity, particular genetic profiles, or specific anatomical characteristics visualized by imaging, could revolutionize the approach to this technique by allowing precise targeting of patients most likely to benefit [59]. Technological evolution continues with the development of increasingly sophisticated devices capable of selective targeting of nerve fibers according to their specific function, distinguishing for example afferent from efferent fibers [60]. Efferent and afferent nerve fibers posess complementary yet distinct physiological functions. Efferent fibers transmit signals from the central nervous system to the kidney, regulating renin release, renal vascular tone, and tubular sodium handling, whereas afferent fibers convey sensory input from the kidney to central autonomic centers, influencing systemic sympathetic outflow [10]. Technological evolution continues with the development of increasingly sophisticated devices capable of selectively targeting these different nerve populations, distinguishing for example afferent from efferent fibers, with comparative studies suggesting differential efficacy between radiofrequency and ultrasound-based ablation techniques [60]. Alternative approaches such as cryoablation or neurotoxin injection are also the subject of active research and could enrich the available technical therapeutic options [61]. Integration of advanced imaging techniques to guide the procedure and optimize targeting of ablation zones also represents a promising avenue [62]. The combinatorial approach, associating RDN, medical optimization, and lifestyle modifications in an integrated therapeutic strategy, could allow maximizing efficacy while personalizing management according to individual patient characteristics. This holistic approach, taking into account not only technical aspects of the procedure but also psychosocial and behavioral dimensions of hypertension, probably represents the future of resistant hypertension management [63].

## 2 Conclusions

RDN marks a significant evolution in the management of resistant hypertension. After a phase of initial enthusiasm followed by a period of skepticism, current data establish its moderate but significant efficacy. By providing durable and kidney-specific sympathetic modulation, RDN complements pharmacologic therapy in selected patients. Recent international guidelines now recognize RDN as a legitimate therapeutic option, while emphasizing the importance of rigorous patient selection and performance in experienced centers. Several challenges persist: optimization of patient selection, technique improvement, long-term evaluation of efficacy and safety. RDN does not constitute a universal solution but represents a valuable therapeutic tool in the management of RH. The future of RDN probably resides in its judicious integration into personalized therapeutic strategies, guided by better understanding of individual pathophysiological mechanisms and predictive response factors. The inclusion of RDN in guidelines marks a major milestone, but the real challenge now lies in its translation into routine clinical practice, with the need to ensure optimal patient selection, adequate operator training, and rigorous long-term follow-up to confirm the cardiovascular benefits suggested by blood pressure reductions observed in clinical trials.

## CRediT authorship contribution statement

**Elena-Mihaela Cordeanu:** Methodology, Formal analysis, Conceptualization. **Emma Morisot:** Formal analysis, Data curation. **François Bronner:** Formal analysis, Conceptualization. **Eric Prinz:** Methodology, Formal analysis, Conceptualization. **Dominique Stephan:** Writing – original draft, Validation, Supervision, Project administration, Methodology, Formal analysis, Data curation, Conceptualization.

## Declaration of generative AI and AI-assisted technologies in the writing process

During the preparation of this work the authors used Claude (Anthropic) in order to assist with manuscript structuring, language editing, and bibliographic formatting. The AI tool was used to help organize the literature review, improve readability, and ensure proper citation formatting. After using this tool, the authors reviewed and edited the content as needed and take full responsibility for the content of the publication. All scientific content, analysis, and conclusions are entirely those of the authors.

## Funding

None declared.

## Declaration of competing interest

The authors declare no conflicts of interest.
